# Article 10 of the Poor-Law Orders

**Published:** 1914-01-31

**Authors:** C. Thackray Parsons

**Affiliations:** Fulham Infirmary


*To the Editor of* The Hospital.

### Article 10 of the Poor-Law Orders.

*To the Editor of* The Hospital.

Sir,—The interesting letter in your last number on Article 10 of the Poor Law Institutions Order, 1913, is unjust to the compilers of that Order. The writer ignores the new powers given to the workhouse medical officer by that Article, and overlooks the fact that the compilers had no authority to alter the law of the land. Under the old Orders the classification of the inmates of the workhouse was left to the Guardians to be carried out in accordance with certain general regulations, and they were only required to *consult* the medical officer when making such arrangements as they might deem necessary with regard to persons labouring under any disease of body or mind. Under Article 10 of the new Order the Guardians *must have regard* to recommendations to be obtained in writing from the medical officer in forming classes to include *infants*, or *persons who are infirm*, or suffering from disease of body or mind, or *women who are pregnant* or *have recently been confined* or *are suckling infants*, and *in assigning accommodation to those classes*. Obviously the Article greatly increases the responsibility and powers of the medical officer. The phrase "must have regard to" is stronger than the word "consult," and the number of inmates for whose classification and accommodation the Guardians cannot provide without the guidance of the medical officer is considerably increased.

With regard to Sub-division (4) of Article 10, this simply embodies the provisions of 10 and 11 Vict. c 109 s. 23 and 39 and 40 Vict. c 61 s. 10. Under the former statute, any married couple in a workhouse both of whom are above sixty are given the right to live together, and under the latter the Guardians are given the power to allow a married couple in a workhouse, one of whom is infirm, sick, or disabled by any injury or above the age of sixty, to live together. No trouble has occurred in the past owing to the provisions of these two statutes, and there seems no reason to anticipate any trouble from them in the future. As regards the removal of sick inmates from the married quarters to the sick wards, the medical officer under the new Order is in exactly the same position as he was under the old Orders. The new Order is evidently being studied by medical officers more carefully than the old Orders and circulars, and they are learning much as regards their responsibilities, powers, and disabilities. That is a happy augury for the future, however troubled the present may appear.

Forsan et haec olim meminisse juvabit;
Durate et vosmet rebus servate secundis.

Yours faithfully,
C. THACKRAY PARSONS.

Fulham Infirmary, January 24, 1914.

